# Treatment of Allergic Rhinitis Is Associated with Improved Attention Performance in Children: The Allergic Rhinitis Cohort Study for Kids (ARCO-Kids)

**DOI:** 10.1371/journal.pone.0109145

**Published:** 2014-10-17

**Authors:** Dong-Kyu Kim, Chae Seo Rhee, Doo Hee Han, Tae-Bin Won, Dong-Young Kim, Jeong-Whun Kim

**Affiliations:** 1 Department of Otorhinolaryngology-Head and Neck Surgery, Chuncheon Sacred Heart Hospital, Hallym University College of Medicine, Chuncheon, Republic of Korea; 2 Department of Otorhinolaryngology-Head and Neck Surgery, Seoul National University College of Medicine, Seoul National University Bundang Hospital, Seongnam, Republic of Korea; 3 Department of Otorhinolaryngology-Head and Neck Surgery, Seoul National University College of Medicine, Seoul National University Hospital, Seoul, Korea, Republic of Korea; University of North Carolina at Chapel Hill, United States of America

## Abstract

**Background:**

It has been well known that pediatric allergic rhinitis was associated with poor performance at school due to attention deficit. However, there were no cohort studies for the effect of treatment of allergic rhinitis on attention performance in pediatric population. Thus, the aim of this study was to investigate whether attention performance was improved after treatment in children with allergic rhinitis.

**Methods:**

In this ARCO-Kids (Allergic Rhinitis Cohort Study for Kids), consecutive pediatric patients with rhinitis symptoms underwent a skin prick test and computerized comprehensive attention test. According to the skin prick test results, the children were diagnosed as allergic rhinitis or non- allergic rhinitis. All of the patients were regularly followed up and treated with oral medication or intranasal corticosteroid sprays. The comprehensive attention tests consisted of sustained and divided attention tasks. Each of the tasks was assessed by the attention score which was calculated by the number of omission and commission errors. The comprehension attention test was repeated after 1 year.

**Results:**

A total of 797 children with allergic rhinitis and 239 children with non-allergic rhinitis were included. Initially, the attention scores of omission and commission errors on divided attention task were significantly lower in children with allergic rhinitis than in children with non-allergic rhinitis. After 1 year of treatment, children with allergic rhinitis showed improvement in attention: commission error of sustained (95.6±17.0 *vs* 97.0±16.6) and divided attention task (99.1±15.8 *vs* 91.8±23.5). Meanwhile, there was no significant difference of attention scores in children with non-allergic rhinitis.

**Conclusions:**

Our study showed that management of allergic rhinitis might be associated with improvement of attention.

## Introduction

Allergic rhinitis (AR) is one of the most common chronic diseases and a global health issue that represents a significant healthcare burden with serious adverse effects on quality of life. The prevalence of AR in adults is estimated to be between 10% and 30%, whereas several studies have estimated the prevalence of AR in children to be closer to 40% [1]–[3]. AR also puts a major socioeconomic burden on patients, their families, health care systems and whole society. The total burden of AR comprises not only indirect costs such as impaired physical and social functioning but also direct financial burdens including use of the health care system and treatment of comorbid diseases to improve patient's health status [4]. One study for socioeconomic burden of AR showed that allergic patients had an approximate two-fold increase in medication costs and 1.8-fold the number of visits to health practitioners [5]. Additionally, in the United States, AR resulted in 3.5 million losses of workdays and 2 million losses of school days annually [5].

Although AR is still considered by many to be a bothersome condition of childhood [5], [6], it may have a considerable effect on quality of life, if left untreated, including sleep disturbance [7]–[9], attention and psychomotor function [10]–[12], participation in social activities [11]–[13], and can result in learning impairment [5], [9], [14]. Furthermore, sleep-disordered breathing in childhood and adolescence is associated with increased disorders of learning performance, behavior, and attention [15]–[18]. However, to date, the effect of treatment for pediatric AR on attention is yet to be investigated using cohort analyses.

Thus, we hypothesized that regular treatment of AR would improve attention. The aim of this prospective study was to investigate whether appropriate treatments were able to improve the attention performance in children with AR by analyzing data collected from children of a large Korean AR cohort.

## Materials and Methods

### Participants

The present study was a longitudinal prospective cohort analysis conducted from February, 2009 through June, 2013, using a database of ARCO-Kids (Allergic Rhinitis Cohort Study for Kids), which is a prospective hospital-based cohort of children with allergic or non-allergic rhinitis (NAR) in Korea. All of the enrolled children received an endoscopic examination and a skin prick test (SPT). Exclusion criteria were as follows: (1) children younger than 3 years or older than 16 years of age; (2) children who had previously been diagnosed with attention deficit hyperactivity disorder or other psychotic disorders; (3) children who had hearing or visual problem; (4) children with adenotonsillar hypertrophy, craniofacial syndromes, or neuromuscular diseases. A written informed consent was obtained from participants or their parents. The study protocol was approved by the Institutional Review Board of Seoul National University Bundang Hospital and Seoul National University Hospital.

### Evaluation of Atopic Status

All of the children underwent SPT for evaluating their atopic status. The SPT was performed by using standardized extracts (Allergopharma, Reinbek, Germany) for 13 common aeroallergens, including house dust mites (*Dermatophagoides pteronyssinus*, *Dermatophagoides farinae*), molds (*Aspergillus fumigatus*, *Alternaria alternata*), animal danders (cat epithelia, dog epithelia), pollens (tree pollen mixture I, tree pollen mixture II, oak, grass pollen mixture, mugwort, and ragweed) and cockroach (*Blattella germanica*). Histamine and isotonic saline were used as positive and negative controls, respectively. The positive result of SPT was diagnosed when the ratio of an allergen to histamine wheal diameter was equal to 1 or greater and the mean wheal diameter of the allergen was 3 mm larger than that of negative (saline) control. Then, the children were categorized into AR and non-AR groups. For children with AR, oral medication and/or intranasal steroid sprays were used regularly for 1 year, whereas for those with non-AR, symptomatic treatment was performed on demand.

### Comprehensive Attention Test

**Figure 1:**
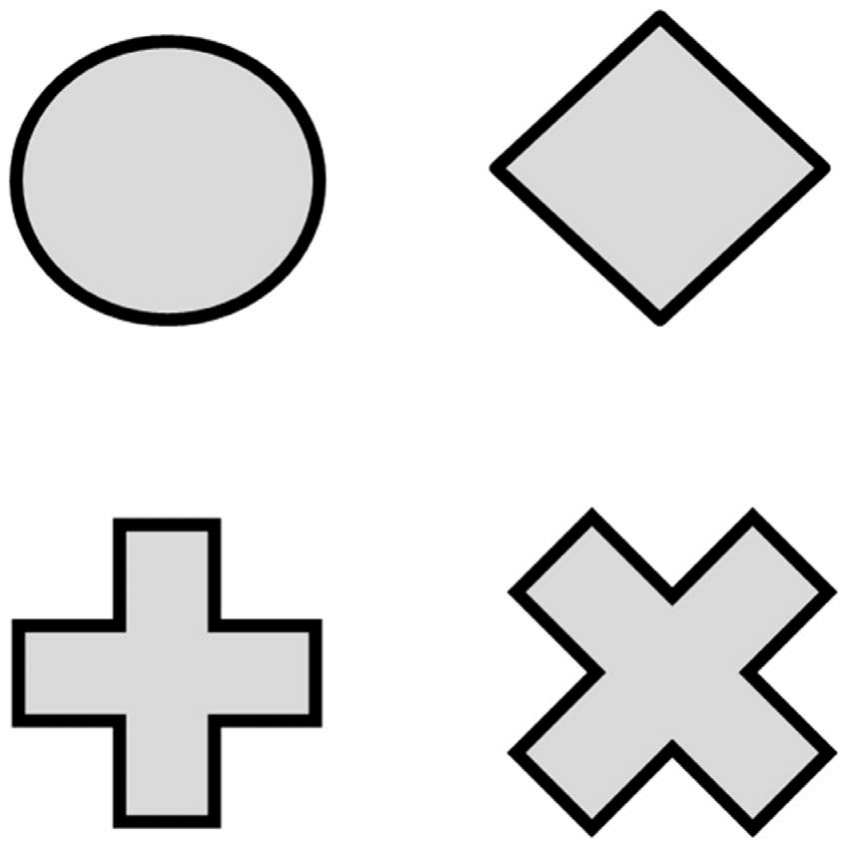
The computerized CAT consisted of tests for sustained attention and divided attention. During sustained attention test, participants were required to respond to a “circle”, “diamond”, “cross” figures except “X” figure.

**Figure 2:**
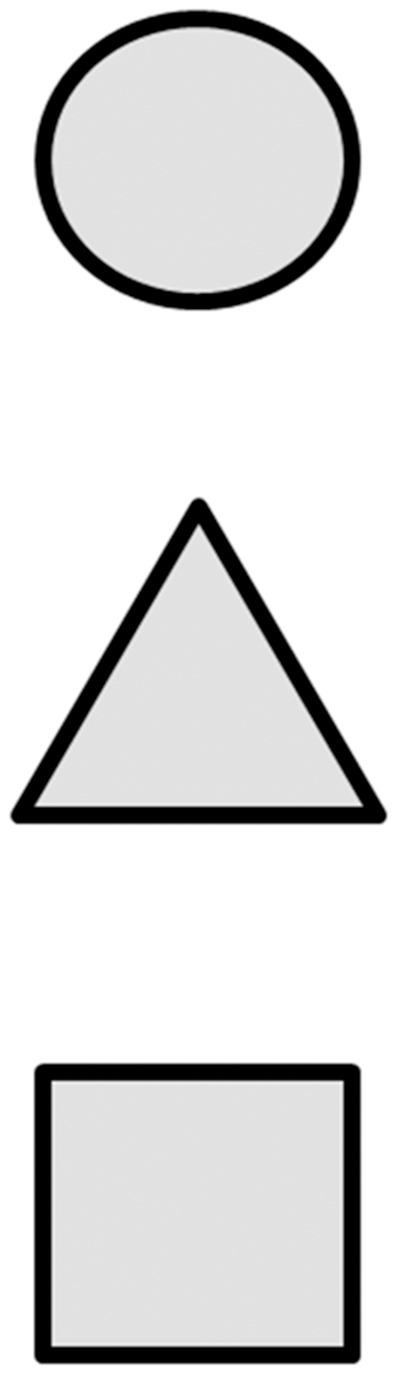
The computerized CAT consisted of tests for sustained attention and divided attention. During divided attention test, participants were required to respond only when prior sounds (bell, camera, buzzer) or previous figures (circle, triangle, quadrangle) were repeated.

The attention status was evaluated by using the computerized comprehensive attention test (CAT), which was made by the Korean Academy of Child and Adolescent Psychiatry [19], [20]. The computerized CAT for sustained attention and divided attention was performed as previously described [19], [20]. Briefly, the sustained attention task assessed the ability to maintain a consistent behavioral response during continuous and repetitive activities. Visual stimuli in various shapes were presented every 2 seconds for 10 minutes. Participants were instructed to respond to all of the shape stimuli except the X shape (Figure 1). Therefore, the task measured the capacity of participants to inhibit responses to certain stimuli under conditions of sustained attention. Meanwhile, the divided attention task demanded more attention than the sustained attention task. Divided attention involved the ability to respond simultaneously to more than two tasks. In the divided attention task, auditory and visual stimuli were presented simultaneously every 2 seconds for 3 minutes 20 seconds, and participants were instructed to respond only when an auditory or visual stimulus was the same as that presented in the preceding pair of stimuli (Figure 2).

In addition, there were two dependent variables (omission and commission errors) on each of the 2 attention tasks. Omission errors were measures of inattention and were defined as failures to respond to the target. Commission errors were measures of impulsivity and were defined as inappropriate responses to the non-target. For removal of potential confounding factors, the resulting values of computerized CAT were presented as the attention score that is an adjusted value based on the standard population who matched gender and age. The standard population showed scores of attention task with mean of 100 and standard deviation of 15.

### Statistical Analysis

The values are presented as mean ± standard deviation (SD) unless otherwise specified. The independent *t*-test was used to determine whether there were associations between allergic rhinitis and inattention. In addition, the paired *t*-test was performed to compare the repeated computerized CAT results. The SPSS statistical software version 18.0 (SPSS Inc, Chicago, IL, USA) was used for statistical analyses. A *P*-value less than 0.05 were considered statistically significant.

## Results

### General characteristics

A total of 1070 consecutive children were enrolled initially in our cohort. Among them, 1036 children (96.8%) who completed CAT at baseline and after 12 months were eligible for analyses in our study. Their mean age was 10.8 years (range, 4–16 years), and 704 participants (67.9%) were males. Among them, 797 children (76.9%) were classified as having AR and 239 children (23.1%) as having non-AR. The general characteristics of the participants are presented in Table 1 according to the type of rhinitis. The mean age was significantly lower in non-AR group (10.1±2.6) than in AR group (11.4±2.6) (p = 0.001). There were no significant differences in gender ratio, body mass index, ARIA severity, and presence of asthma between the two groups.

**Table 1:** General characteristics of subjects.

	Allergic rhinitis	Non-allergic rhinitis	p value
	(N = 797)	(N = 239)	
Age, years, mean (SD)	11.4 (2.6)	10.1 (2.6)	0.001
Gender (male), n (%)	561 (70.4%)	163 (68.2%)	0.521
BMI, kg/m^2^, (z-score)	0.32±2.1	0.29±3.1	0.157
ARIA classification, n (%)			0.051
*Mild intermittent*	149 (18.7%)	64 (26.8%)	
*Moderate/severe intermittent*	144 (18.1%)	43 (18.0%)	
*Mild persistent*	87 (10.9%)	23 (9.6%)	
*Moderate/severe persistent*	417 (52.3%)	109 (45.6%)	
Asthma, n (%)	139 (17.4%)	32 (13.4%)	0.164

### Baseline Attention Status According to the Presence of Allergic Rhinitis

According to the presence of AR, the initial attention status was evaluated using the CAT. On the sustained attention task of non-AR group, the mean attention score of omission errors and commission errors was 98.3±17.8 and 98.2±17.3, respectively. Meanwhile, AR group had a 99.9±19.3 of omission errors and a 95.6±17.0 of commission errors on the sustained attention task. Although AR group children made more commission errors than non-AR group children on the sustained attention task, the differences were not statistically significant (p = 0.056).

On the divided attention task, the mean attention score of omission errors and commission errors in non-AR group was 100.4±23.1 and 99.1±15.8, respectively, whereas AR group showed a 93.9±16.4 of omission errors and a 91.8±23.5 of commission errors. AR group children made significantly lesser omission errors (p = 0.022) and commission errors (p = 0.046) on the divided attention task than non-AR group children.

### Effect of Treatment on Attention Status

To investigate the treatment effect on attention status in each group, we compared the mean attention score of CAT between baseline and after 1 year. Compared to pre-treatment attention performance, AR group showed significantly higher attention score of commission errors in both sustained attention task (p = 0.003) and divided attention task (p = 0.011) (Table 2), but there was no significant difference in omission errors in both sustained and divided attention tasks. On the contrary, Non-AR group showed no significant differences in the mean attention scores of omission errors and commission errors in both sustained and divided attention tasks (Table 3).

**Table 2:** Comparisons of comprehensive attention test results between pre- and post-treatment in children with allergic rhinitis.

Allergic rhinitis	Before treatment	After 1 year treatment	p value
(N = 797)			
***Sustained-attention test (SD)***			
Omission errors	99.9±19.3	100.3±17.8	0.861
Commission errors	95.6±17.0	97.0±16.6	0.003
***Divided-attention test (SD)***			
Omission errors	93.9±16.4	96.4±21.2	0.603
Commission errors	91.8±23.5	96.3±19.8	0.011

**Table 3:** Comparisons of comprehensive attention test results between pre- and post-treatment in children with non-allergic rhinitis.

Non-allergic rhinitis	Before treatment	After 1-year treatment	p value
(N = 239)			
***Sustained-attention test (SD)***			
Omission errors	98.3±17.8	98.2±20.0	0.344
Commission errors	98.2±17.3	96.1±15.8	0.470
***Divided-attention test (SD)***			
Omission errors	100.4±23.1	99.0±21.3	0.071
Commission errors	99.1±15.8	101.6±29.6	0.615

## Discussion

Pediatric AR is a common debilitating disorder that can adversely affect the quality of life and the academic performance of school-age children. The overall socioeconomic burden of AR is a great expense, with direct expenditures, such as costs of physician visits and medications. Moreover, indirect costs of this disease including missed school days, decreased learning capacity, reduced school performance, impaired sleep, and diminished quality of life are substantial [5], [21]. However, although the overall cost of AR in children is considerable, its impact is often underestimated [12].

In the present study, we found that children with AR showed a worse performance on divided attention compared to children with non-AR. In addition, in terms of commission errors of sustained/divided attention, the partial improvement of attention status was observed by treatment in pediatric AR. Our findings could be supported by previous studies showing that treatment of pediatric AR improved learning ability [14], [22]. The study by Allergic Rhinitis in School children Consensus Group stated that treatment with non-sedating second-generation antihistamine has been shown to improve learning potential [14]. A case-control study also showed that the seasonal AR reduced learning ability of children but this effect was partially counteracted by treatment of AR [22]. To our knowledge, this is the first study to examine the relationship between treatment of allergic rhinitis and performance of sustained/divided attention in children using objective measures.

Sustained attention means the ability to direct and focus cognitive activity on specific stimuli [23], [24]. Thus, in order to complete any cognitively planned activity or any thought, one must use sustained attention. An example is the act of reading a newspaper article. One must be able to focus on the activity of reading long enough to complete the task. Meanwhile, divided attention is the process of actively paying attention to two or more tasks at the same time [25], [26]. An example is the act of reading an email while listening to someone talk at the same time or watching television while surfing on internet simultaneously. Commission errors are defined as inappropriate responses to the non-target, whereas omission errors are defined as some mistakes that occur when action has not been taken or when something has been left out. Nasal obstruction and rhinorrhea are characteristic symptoms that have the greatest impact on sleep, since postnasal drip and mucosal edema obstruct the nasal cavities, increasing nasal resistance [7], [27], [28]. Some studies based on preschool-aged children showed that sleep problems were associated with an increased risk of learning, behavioral, and attention problems [29]–[31]. Moreover, pediatric sleep disordered breathing is related to attention deficit [32], [33]. Previous studies suggested that intermittent hypoxia and sleep disruption from respiratory events may have neurocognitive consequences [34]–[38]. In addition, Findings in some studies reported that sleep deprivation was related to changes in neural structure [39]–[41]. Therefore, sleep disturbance might be one of the mechanisms causing altered attention status in pediatric AR.

Because attention deficit is known to be multifactorial, the present study was designed to examine independent therapeutic effects of AR on attention by using the attention score, adjusting for potential confounding factors, such as gender and age. Based on our results, the appropriate treatment of AR is beneficial for improvement of attention, especially on commission errors of sustained or divided attention tasks. Meanwhile, the therapeutic effect did not appear on omission errors. Our different findings may be explained by prior several studies [42]–[45]. A study for behavioral performance of children described that the commission errors were correlated with hyperactivity–impulsivity. Meanwhile, the omission errors were associated with inattention of caregiver [42]. In addition, studies for attention-deficit/hyperactivity disorder also found that children with atopic diseases had a substantially increased rate of attention-deficit/hyperactivity disorder [43]–[46]. Moreover, some studies for AR children showed that AR in preschoolers was associated with psychological/behavioral problems and untreated AR may contribute to the development of hyperactivity–impulsivity in children [47], [48].

Non-AR has similar symptoms of AR but no identified allergic reaction [49]. To date, the knowledge of pathophysiology in non-AR has been limited and thus, the diagnosis of this condition is made by exclusion [49]. Generally, treatment of non-AR depends on their symptoms, but the control of symptoms using oral medication and/or intranasal steroid sprays may be less effective in non-AR patients than in AR patients [50], [51]. In the present study, we found that the treatment effect on attention was more effective in pediatric AR patients compared with children with non-AR. These data are in agreement with previously reported relatively ineffective treatment outcome in non-AR.

However, our study has some limitations. Firstly, the present study has no healthy control group. Therefore, it is hard to differentiate whether the improvement of attention was the effect of AR treatment or natural improvement by aging. However, non-AR patients who were set as a disease control group did not show improvement. Secondly, there may be a selection bias because our cohort was a hospital-based one. However, it may have been partially overcome because the present study was prospectively performed. Thirdly, our study lacked some important factors such as socioeconomic status and parental education levels that may influence the attention status of children. In spite of these limitations, the advantages of the present study may be its large sample size, the use of objective computerized attention test and a longitudinal design.

## Conclusions

The present study suggests that AR be associated with attention problems compared to non-AR in children. The commission errors of sustained and divided attention tasks could be improved by treatment of AR. Therefore, regular treatment of AR might also be warranted, in particular, in terms of improvement of attention problems in children with AR.
